# Improved balance in middle-aged adults after 8 weeks of a modified version of Otago Exercise Program: A randomized controlled trial

**DOI:** 10.1371/journal.pone.0235734

**Published:** 2020-07-15

**Authors:** Rana Almarzouki, Gurinder Bains, Everett Lohman, Bruce Bradley, Todd Nelson, Samiah Alqabbani, Asma Alonazi, Noha Daher

**Affiliations:** 1 Department of Physical Therapy, School of Allied Health Professions, Loma Linda University, Loma Linda, California, United States of America; 2 Department of Physical Therapy, Faculty of Medical Rehabilitation Sciences, King Abdulaziz University, Jeddah, Kingdom of Saudi Arabia; 3 Department of Allied Health Studies, School of Allied Health Professions, Loma Linda University, Loma Linda, California, United States of America; 4 Department of Rehabilitation Sciences, School of Health and Rehabilitation Sciences, Princess Nourah bint Abdulrahman University, Riyadh, Kingdom of Saudi Arabia; 5 Department of Physical Therapy, School of Applied Medical Sciences, Majmaah University, Riyadh, Kingdom of Saudi Arabia; Texas A&M University, UNITED STATES

## Abstract

**Objective:**

The objective of this randomized controlled trial was to examine dynamic balance changes (reach distance) in middle-aged adults using the Y Balance Test (YBT) following 8 weeks of home-based exercise program adapted from the Otago Exercise Program (OEP).

**Methods:**

Fifty-two healthy middle-aged adults with mean age of 54.4±5.4 years and body mass index of 27.7±5.7 kg/m^2^ were randomly assigned into either the exercise group (a modification of the Otago Exercise Program, that consisted of home-based balance and strength exercises) or the non-exercise group (continuation of usual lifestyle) by having the participants select a paper from a sealed envelope. The YBT was used to measure participants’ dynamic balance in the right and left anterior (RA, LA), posteromedial (RPM, LPM), and posterolateral (RPL, LPL) directions.

**Results:**

The outcome in this trial was reach distance (cm). There was a significant group by time interaction in terms of reach distance for all directions (p<0.05, η^2^ ranged from 0.06 to 0.20). In the exercise group, results of the repeated measures analysis of variance (ANOVA) showed significant improvements in the reach distance in all the directions (p<0.001). In contrast, the non-exercise group had significant difference only in the left posterolateral direction (p = 0.009). Participants in the exercise group achieved significantly greater reach distance (cm) (95% confidence interval (CI)) for RA[(2.8, 0.4 to 5.2), p = 0.023]; LA[(3.2, 0.9 to 5.6), p = 0.008]; RPM[(4.0, 1.0 to 7.9), p = 0.046]; LPM[(5.8,1.3 to 10.3), p = 0.013]; RPL[(7.6, 2.6 to 12.6), p = 0.003]; and LPL[(4.2, 0.3 to 8.2), p = 0.035].

**Conclusion:**

The modified version of OEP appears to be effective in improving parameters of dynamic balance in the middle-aged adult population. The improvements in YBT reach distance in the exercise group are indicative of the significance of performing balance and strength exercises regularly for this population.

## Introduction

Falls can present numerous negative health outcomes for middle-aged adults, and further, has been identified as the third leading cause of unintentional injury deaths in Americans aged 45–64 years [1]. Additional consequences of such falls may include serious hospitalization, decline in quality of life, injury, or death [2]. A previous investigation found that middle aged and older adults experienced one to two falls during the two year period, (21% in middle-aged adults and 35% in older adults), and most falls occurred in their homes [3]. Caban-Martinez et al. (2015) observed that 18% of middle-aged adults had experienced at least one fall in the past three months [1]. The number of fall-induced deaths was 29,759 and 3,917 in adults over the age of 60 years and middle aged-adults, respectively [4]. In addition, hospitalization costs in 2010 were over $25 billion and $7 billion, respectively, in both groups [5, 6].

For decades, researchers have focused on investigating the causes of falling in older adults. Dynamic balance and leg muscle strength have a profound relation to fall risk [7]. Therefore, improving such factors may reduce the frequency of falling and its costs [7]. Daley et al. (2013) recommended improving balance as early as the age of 60 years [8]. Ferreira et al. (2012) concluded that exercise programs that include strength and balance may prevent future falls in adults aged 40–60 years [9].

Balance, which declines with aging, is a complex sensory-motor process in which the visual, vestibular, and musculoskeletal systems work cooperatively to produce postural stability [10, 11]. Additionally, proprioception, the neural input from the mechanoreceptors distributed throughout the human body, deteriorates with aging as well and affects balance, thus increasing the risk of fall [12]. Moreover, aging adversely affects the joints structures as a result; performance of physical activities would be affected negatively [12].

The Otago Exercise Program (OEP) is an exercise program designed to prevent falls among older adults. It is a home-based exercise program that consists of a walking plan in addition to strengthening and balance training exercises that are personalized according to one’s needs [13]. Ambrose et al. (2008) investigated the effect of the OEP on adult fallers who were 70 years and older [14]. Those who received OEP experienced improvements in cognitive functioning and reduction in the incidence of falls [14]. Yoo et al. (2013) found that older females who performed the OEP had improvements in gait and balance [15]. As for middle-aged adults, a modification to this program to challenge muscular strength and activity level might be needed to attain the same goal.

In an effort to accurately assess dynamic balance, which is defined as the ability to maintain a stable base of support during movement [16], investigators have developed numerous assessment tools. One of these assessment tools is the Y Balance Test (YBT). The YBT is a valid and reliable test of dynamic balance and does not demonstrate a ceiling effect as compared to other assessment tools such as the Berg Balance Scale [10, 17]. Furthermore, the YBT might be more suited for active adults as it is more challenging than other tests [10]. The YBT involves maintaining a stable unilateral base of support on the platform while the other leg is reaching in the anterior (A), posteromedial (PM), and posterolateral (PL) directions [18]. Lee et al. (2015) reported that the YBT reach distance and lower limb strength was significantly lower in older females compared to the middle-aged females [10]. Similarly, Freund et al. (2015) indicated that healthy females aged 50–59 years had better reach distance than females 60–79 years of age [18]. This implies that dynamic balance decreases with age [10]. Furthermore, Lee et al. (2014) reported a positive correlation between participants’ performance in all YBT directions and hip extensor strength [19].

In the available literature, no studies have examined the effect of a home-based exercise program derived from the OEP on balance in middle-aged adults. Therefore, this study aimed to examine dynamic balance changes in middle-aged adults using the YBT following 8 weeks of a modified version of the Otago Exercise home Program. The primary outcome measure for the study was reach distance. The reach distance was compared between participants who completed the exercise program and those in the non-exercise group in all YBT directions. We hypothesized that: 1) the reach distance in all directions among participants in the exercise group will significantly improve overtime; 2) the reach distance in all directions among participants in the non-exercise group will not change between baseline and 8 weeks; and 3) the reach distance among participants in the exercise group would be significantly higher than that in the non-exercise group.

## Methods

### Participants

**Fig 1:**
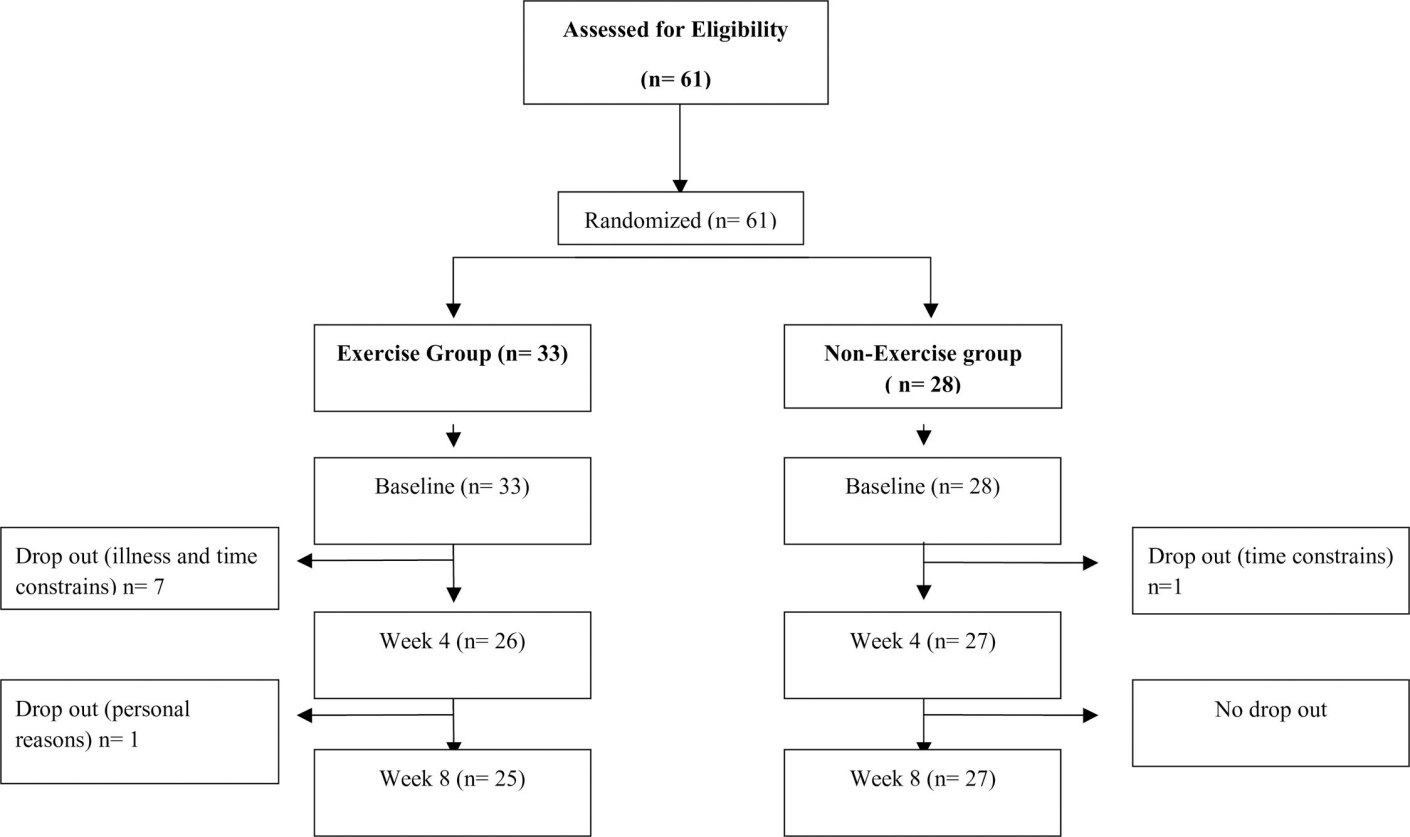
Consort flow diagram of participants’ recruitment and retention.

Participants in this study were healthy adults between the age of forty-five and sixty-four years, and able to walk and move independently. Participants were excluded if they had dizziness, visual or neurological disorders that impaired their ability to complete the testing protocol, lower limb injury in the last three months, lower limb surgery in the last six months, or a current involvement in a rehabilitation program. Participants were recruited from Southern California by distributing flyers, word of mouth, emails, and social media. Sixty-one participants were screened and tested at baseline, with an attrition of nine participants due to illness and personal reasons. Fifty-two participants completed the study (Fig 1). This study was approved by the Institutional Review Board of Loma Linda University, Loma Linda, California on 07/01/2016 and was completed on 06/01-2019. The study protocol was explained to the participants and all signed the informed consent. This study was registered in ClinialTrails.gov with a protocol # 03161236. When this study began on October 31, 2016, this study did not meet the definition of an Applicable Clinical Trial (ACT) and as such, was not required by FDAAA 801 to be registered on ClinicalTrials.gov. In reviewing the ICMJE website, it appears that ICMJE first published their ClinicalTrials.gov recommendation in December 2016, which is two months after the first human subject was enrolled on this specific study. The study was registered on ClinicalTrials.gov as soon as our department became aware of the ICMJE policy in March 2017. Since March 2017, in conjunction with our Human Research and Compliance Department, we have developed specific education for Principal Investigators and their students, which address both federal requirements for clinical trials registration and ICMJE requirements for publication. The first participant was run on 1010/31/2016 and the last visit of the last participant was on 12/20/2017. The authors confirm that all ongoing and related trials for this intervention are registered.

### Dynamic balance instrumentation

#### Y balance test—lower quarter

Y Balance Test (YBT) (FunctionalMovement.com, Danville, VA) was used to measure dynamic balance as it assesses performance during single-leg balance with a reaching task in the anterior (A), posteromedial (PM), and posterolateral (PL) directions to determine lower extremity movement asymmetry and balance deficits [20]. The test has a standardized protocol with well-defined pass/fail criteria. The test has high intrarater reliability: ICC ranged from 0.85–0.91, and interrater reliability: ICC ranged from 0.99–1.00 [17]. The device consists of a wooden platform on which participants stand with a single leg while the other leg is placed on the floor behind the bar. Three wooden bars with one-centimeter increment markings for score recordings are attached to the central stance platform in the three testing directions (A, PM, and PL). The two posterior bars are positioned 135° from the anterior bar and 90° from each other. On the top surface of the three bars, a reach indicator with a red target on the side where the participant pushes is placed for reaching in the three desired directions. The reach distance is recorded from the edge of the indicator at the nearest point to the platform.

### Physical activity level instrumentation

#### The rapid assessment of physical activity questionnaire

Participant’s activity level was determined using the Rapid Assessment of Physical Activity Questionnaire (RAPA). This is a nine-item self-administered questionnaire. It examines aerobic (RAPA I), strength, and flexibility (RAPA II). According to the scoring, the participants were classified in the different aerobic categories ranging from sedentary to active. Moreover, the scores determined the strength and flexibility levels. The University of Washington Health Promotion Research Center developed this questionnaire and permission was granted. The validity of this questionnaire is r = 0.45 with a test-retest reliability: ICC = 0.65 [21]. The questionnaire was explained to the participants.

### Home-based exercise program

The exercise program was intended to improve balance and strength. It was adapted from the Otago Exercise Program that was designed for older adults [13]. The modification and design of the current exercise program were completed by a group of physical therapy clinical specialists in orthopedics, geriatrics, and neurology. One of the specialists is YBT certified. All the exercises were completed at the participants’ ability level. Participants were not instructed in specific footwear while doing the exercises. The program took up to 15 minutes to complete and was repeated three times a week for eight weeks. A single investigator taught all the exercises to ensure that all participants received the same instructions and to ensure that they understood and accurately executed the exercises.

The prescribed program consisted of three exercises for balance and two strengthening exercises. The first balance exercise was “standing on one foot.” Participants were asked to stand on one foot holding on to a stable surface as needed, and progress to stand on one foot for one minute without holding. This exercise was repeated two times per leg for a total of four minutes (as opposed to the 30 seconds used in the original Otago program) throughout the eight-weeks period. If the participant was not able to stand for a minute, they continued at their own pace to accomplish the goal of the exercise and then they continued with the rest of the exercise program. The second balance exercise was “walking heel to toe.” Participants were asked to walk with the heel of one foot touching the toes of the other foot. This exercise was performed for twenty repetitions in both forward and backward directions in the first four weeks; the repetitions progressed to forty times in both directions in the second four weeks (as opposed to the original Otago which starts with ten repetitions with hold for support to progress to no hold). The third balance exercise was “heel walking.” Participants were instructed to walk on their heels with the same number of repetitions and progression as the “walking heel to toe exercise” (whereas the original Otago includes heel walking in the forward direction only). For the last two balance exercises, participants were asked to perform them next to a wall in order to prevent falling.

There were two strengthening exercises. The first strengthening exercise was “side-kicks.” Participants, while standing, were directed to raise their leg out straight to the side while holding on a stable surface without leaning. A red Theraband was wrapped around their mid calves to provide light resistance in the first four weeks. Participants repeated this exercise for twenty repetitions per leg. A blue Theraband was used for heavier resistance in the second four weeks with a progression to forty repetitions. One to two minutes of rest was given between exercising each leg, which differs from the original Otago that uses ankle cuff weights. The second strengthening exercise was “wall-squats.” This exercise was included as an additional exercise to the original Otago exercise program to fit the functional level of the middle-aged participants as opposed the older adults. The participants were requested to slide down on a wall to a sitting position with their ankles placed under their knees in a perpendicular manner to avoid knee injury. Once the participant reached the sitting position with their knees and hips at 90 degrees of flexion, they returned to the erect standing position and performed the same exercise in a continuous fashion for twenty repetitions for the first four weeks and up to forty repetitions for the second four weeks. Exercise description is summarized in (Table 1).

**Table 1:** Exercise description.

Exercise Group	First 4 weeks	Second 4 weeks
	***Balance Exercises***	***Balance Exercises***
	**1- Standing on one foot:** Up to 1 minute	**1- Standing on one foot:** Up to 1 minute
	**2- Walking heel to toe:** 20 repetitions forward & backward	**2- Walking heel to toe:** 40 repetitions forward & backward
	**3- Heel walking:** 20 repetitions forward & backward	**3- Heel walking:** 40 repetitions forward & backward
	***Strengthening Exercises***	***Strengthening Exercises***
	**1- Side-kicks:** 20 repetitions with red Theraband.	**1- Side-kicks:** 40 repetitions with blue Theraband.
	**2- Wall-squats:** 20 repetitions	**2- Wall-squats:** 40 repetitions
**Non-Exercise Group**	Continued with usual lifestyle	

## Procedures

### Leg length measurement

Lower limb length was measured in order to normalize YBT reach distance. With the participant lying supine on a plinth, the participant was asked to perform a bridge by lifting the hips from the plinth. The investigator passively straightened the legs. Using a measuring tape, the lower limb length was recorded in centimeters from the inferior aspect of anterior superior iliac spine to the most distal portion of the medial malleolus. The same investigator took all the measurements.

### Y balance test—lower quarter

Prior to testing, participants watched the Y Balance Test Lower Quarter Client Instruction video (FunctionalMovement.com, Danville, VA). Next, the investigator demonstrated the movements and then the participant practiced each movement six times on the floor barefoot. Three minutes of rest were given to the participant to reduce fatigue prior to the actual test. On the YBT platform, the investigator recorded three successful readings in each direction. No instructions were given regarding hand placement during the test. The investigator and a research assistant stood next to the participant for safety. The testing protocol consisted of the following directions: right anterior (RA), left anterior (LA), right posteromedial (RPM), left posteromedial (LPM), right posterolateral (RPL), and left posterolateral (LPL) [17]. A testing trial was discarded and repeated if the participant lost balance or stepped off the platform, supported the reaching foot on top of the indicator to gain support, was unable to keep the reaching foot in close contact with the indicator while reaching, or was incapable of returning the reaching foot to the starting position [17].

### Execution of protocol

After signing the consent form, the investigator collected the participant’s weight, height, age, hand and leg dominance that were determined based on participant’s report, and lower limb length measurement. Participants’ group allocation to exercise or non-exercise groups was randomly determined at baseline by having the participants select a paper from a sealed envelope. Participants in the exercise group received an exercise packet with the exercise instructions provided in a written form and a DVD that had a video of the exercises along with log sheets and Therabands. Participants were requested to perform the exercise program in addition to their usual routine. Each participant in the exercise group was contacted on a weekly basis to ensure his/her compliance with the exercise protocol and address any concerns. Also, they were required to record the number of repetitions per each exercise and the exercise day. Participants were instructed to continue with their usual lifestyle if they were in the non-exercise group. Participants filled out the RAPA questionnaire at baseline only. Participant’s balance was measured using YBT at baseline, four weeks later, and eight weeks later. At the completion of the study, exercise packets and instructions were provided to the non-exercise group participants. Participants’ balance was measured using the Limits of Stability Test (LOS) at the three visits and the testing order of the YBT and LOS was randomly determined. Also, the Pittsburgh Sleep Quality Index (PSQI) and Short Form-36 (SF-36) were filled out at baseline and eight weeks visits. The results of the LOS, PSQI, and SF-36 will be published in a different manuscript.

### Data analysis

For data analysis, the reach distance was normalized to the lower limb length by using the maximum reach distance in each direction using the following formula: (maximum reach distance/ limb length) * 100 [17].

### Statistical analysis

Data analysis was performed using SPSS Statistics Software version 24.0 (IBM Corp, Armonk, NY). A sample size of 73 subjects was estimated using a medium effect size (partial η^2^ = 0.06), a power of 0.95, level of significance set at 0.05, and a 40% dropout.

Mean ± standard deviation (SD) was computed for continuous variables and frequencies (%) for categorical variables. Normality of quantitative variables was assessed using Shapiro-Wilk test and boxplots. We compared mean age (years), Body Mass Index (kg/m^2^) (BMI), and reach distance (cm) in all directions at baseline between the two groups using independent t-test. Distribution of qualitative variables (gender, dominant leg, RAPA I, and RAPA II) by group type was examined using Chi Square test (Table 2).

**Table 2:** Frequency (%) of general characteristics by study group (N = 52).

Characteristics	Exercise (n_1_ = 25)	Non-Exercise (n_2_ = 27)
**Female**	21 (84)	21 (77.8)
**Age (years); mean (SD)**	53.9 (5.0)	55.0 (5.8)
BMI (kg/m^2^); mean (SD)	27.1 (5.9)	28.4 (5.5)
**RAPA I**		
Underactive	1 (4.0)	2 (7.4)
Underactive regular-light activities	2 (8.0)	1 (3.7)
Underactive regular	0 (40.0)	6 (22.2)
Active	2 (48.0)	18 (66.7)
**RAPA II**		
Neither	6 (33.3)	12 (63.7)
Strong	3 (0.75)	1 (0.25)
Flexible	9 (0.75)	3 (0.25)
Both	7 (38.8)	11 (61.2)

**Abbreviation:** SD, Standard Deviation; RAPA, Rapid Assessment of Physical Activity Questionnaire.

To compare the reach distance for the three YBT directions (right and left side) by study group (exercise versus non-exercise) over time (baseline versus 4 weeks versus 8 weeks) a general linear model (GLM) was conducted. Comparison between the two groups was done using the group x time interaction effect. If the interaction was statistically significant, a One-way repeated measures ANOVA was conducted to determine changes in the reach distance for each group separately. If the results of the repeated measures ANOVA were significant, post hoc comparisons using Bonferroni adjustment and effect sizes were computed to identify significant differences. Analysis of covariance was used to control for the effects of gender and age. We also computed the change in reach distance (cm) from baseline to eight weeks and compared it between the two groups using independent t-test. The level of significance was set at *p*≤ 0.05.

## Results

Sixty-one participants were recruited originally for this randomized controlled trial. At week 4 follow-up, eight participants (7 from the exercise group and 1 from the non-exercise group) withdrew from the study due to illness and time constraints (Fig 1). At the week 8 follow-up, one participant dropped out from the exercise group for personal reasons. However, there were no significant differences in the baseline data between participants who dropped out of the study and those who completed the study (p>0.05). In addition, none of the participants reported receiving any additional rehabilitation intervention during the study period.

Fifty-two participants with mean age of 54.4±5.4 years and BMI of 27.7±5.7 kg/m^2^ completed the study. Most of the participants were females (n = 42, 80.8%) and right leg dominant (n = 48, 92.3%). Twenty-five participants (48.1%) were in the exercise group and 27 (51.9%) in the non-exercise group. Frequency (%) of gender and physical activity level and mean (SD) of age and BMI at baseline by study group is displayed in Table 2. In the exercise group, the median compliance rate was 100% (minimum = 75%, maximum = 100%).

All analyses were performed using intention to treat principle, where all participants who were randomized were included in the statistical analysis and analyzed according to the group they were originally assigned. Thus, all 61 participants were included in the analysis. To deal with the missing data for the dropped out participants, we used their last recorded data for the proceeding analysis [22].

### Between–group analysis

Results of the GLM are summarized in Table 3. There was a significant group by time interaction effect for all YBT directions (right and left side) except for RPM (p<0.05, η^2^ ranged from 0.06 to 0.20). Results of the independent t-test showed that there was a significant difference in the change in reach distance (baseline versus 8 weeks later) between the two groups (p<0.05) (Table 4). When we controlled for the effects of gender and age, similar findings were obtained.

**Table 3:** Mean (SD) of reach distance by study group over time (N = 61).

	Exercise Group (n_1_ = 25)				Non-Exercise Group (n_2_ = 27)				
Direction	Baseline	4 weeks	8 weeks	Within group	Baseline	4 weeks	8 weeks	Within group	Group x time
				p-value (η^2^) *				p-value (η^2^) *	p-value (η^2^) **
**RA**	61.2 (6.9)	65.0 (7.7)	65.2 (7.5)	<0.001 (0.30)	60.8 (7.2)	60.6 (7.0)	61.6 (6.2)	0.525 (0.02)	0.007 (0.12)
**LA**	62.7 (6.8)	64.9 (7.8)	66.5 (7.3)	<0.001 (0.32)	60.4 (6.6)	60.2 (6.5)	61.0 (6.5)	0.565 (0.02)	0.012 (0.07)
**RPM**	91.5 (12.6)	96.4 (10.7)	98.6 (10.4)	<0.001 (0.33)	92.8 (12.4)	94.7 (14.1)	95.8 (10.6)	0.107 (0.08)	0.101 (0.04)
**LPM**	94.6 (13.1)	100.4 (11.4)	102.9 (12.3)	<0.001 (0.42)	97.3 (12.0)	97.5 (11.7)	98.3 (11.0)	0.767 (0.01)	0.001 (0.20)
**RPL**	88.5 (13.5)	94.5 (11.7)	97.0 (12.5)	<0.001 (0.48)	95.3 (16.0)	95.2 (12.5)	96.2 (13.0)	0.815 (0.01)	0.002 (0.10)
LPL	89.8 (15.4)	96.1 (13.2)	98.3 (15.1)	<0.001 (0.41)	92.7 (11.9)	94.5 (13.5)	96.9 (11.3)	0.01 (0.16)	0.033 (0.06)

**Abbreviation:** SD, Standard Deviation; RA, Right Anterior; LA, Left Anterior; RPM, Right Posteromedial; LPM, Left Posteromedial; RPL, Right Posterolateral; LPL, Left Posterolateral; η2, Partial Eta Square effect size; d, **η**^**2**^ = $\frac{Treament\;Sum\;of\;Squares}{Total\;Sum\;of\;Squares+Error\;Sum\;of\;Squares}$

*One -Way Repeated Measures ANOVA

**General Linear Model, p≤0.05

**Table 4:** Mean (SD) change with 95% CI in reach distance (cm) from baseline to week eight by study group (N = 61).

Direction	Exercise Group (n_1_ = 25)	Non-exercise Group (n_2_ = 27)	Mean Difference (95% CI)	p-value*	Effect Size (d)*
**RA**	3.6 (4.4)	0.8 (5.0)	2.8 (0.4, 5.2)	0.023	0.59
**LA**	3.8 (4.6)	0.6 (4.6)	3.2 (0.9, 5.6)	0.008	0.69
**RPM**	7.1 (8.1)	3.1 (7.0)	4.0 (1.0, 7.9)	0.046	0.52
**LPM**	11.4 (8.4)	5.6 (9.3)	5.8 (1.3, 10.3)	0.013	0.65
**RPL**	8.6 (8.7)	0.9 (10.7)	7.6 (2.6, 12.6)	0.003	0.78
**LPL**	8.5 (9.3)	4.3 (5.9)	4.2 (0.3, 8.2)	0.035	0.53

**Abbreviations:** SD, Standard Deviation; CI, Confidence Interval; RA, Right Anterior; LA, Left Anterior; RPM, Right Posteromedial; LPM, Left Posteromedial; RPL, Right Posterolateral; LPL, Left Posterolateral; d, Cohen’s d effect size

*Independent t-test

### Within-group analysis

Results of the one-way repeated measures ANOVA are presented in Table 3. For the exercise group, there was a significant change in RA reach distance over time (<0.001, η^2^_=_ 0.30). Bonferroni post hoc comparison revealed that the change was significant between baseline and week 4 (p = 0.001) and between baseline and week 8 (p<0.001). However, there was no significant difference between week 4 and week 8 (p = 1.0). There was also a significant change in LA reach distance over time (<0.001, η^2^ = 0.32). Bonferroni post hoc comparison revealed that the change was significant between baseline and week 4 (p = 0.032) between baseline and week 8 (p<0.001) and between week 4 and week 8 (p = 0.038). For the non-exercise group, there was no significant change in RA and LA reach distances over time (p = 0.525, p = 0.565, respectively).

For the exercise group, there was a significant change in RPM reach distance over time (<0.001, η^2^ = 0.33). Bonferroni post hoc comparison revealed that the change was significant between baseline and week 4 (p = 0.004) and between baseline and week 8 (p<0.001). However, there was no significant difference between week 4 and week 8 (p = 0.133). There was also a significant change in LPM reach distance over time (<0.001, η^2^ = 0.42). Bonferroni post hoc comparison revealed that the change was significant between baseline and week 4 (p<0.001) between baseline and week 8 (p<0.001) and between week 4 and week 8 (p = 0.024). For the non-exercise group, there was no significant change in RPM and LPM reach distances over time (p = 0.107, p = 0.767, respectively).

Lastly, for the exercise group, there was a significant change in RPL reach distance over time (<0.001, η^2^ = 0.48). Bonferroni post hoc comparison revealed that the change was significant between baseline and week 4 (p = 0.002), and between baseline and week 8 (p<0.001). However, there was no significant difference between week 4 and week 8 (p = 0.070). There was also a significant change in LPL reach distance over time (<0.001, η^2^ = 0.41). Bonferroni post hoc comparison revealed that the change was significant between baseline and week 4 (p<0.001), between baseline and week 8 (p<0.001), and between week 4 and week 8 (p = 0.044). For the non-exercise group, there was no significant change in RPL reach distance over time (p = 0.815). However, there was a significant change in LPL reach distance over time (p = 0.009, η^2^ = 0.16). Bonferroni post hoc comparison revealed that the change was significant between baseline and week 8 (p = 0.002), but not between baseline and week 4 (p = 0.714).

## Discussion

This study intended to evaluate a modified version of Otago Exercises Program that was designed to specifically target and improve dynamic balance outcomes. The prescribed program was intended to be safe for middle-aged adults to perform daily without professional supervision. This study was the first study to our knowledge that examined the sensitivity of YBT in detecting balance changes following a modified version of OEP.

The findings of this study support our hypotheses. Results showed that the exercise group had significant improvements in YBT reach distance in all reaching directions overtime. In contrast, the non-exercise group showed an improvement in the LPL between baseline and 8 weeks. Nonetheless, when comparing change in reach distance from week 8 to baseline between the two study groups, the change in all directions was significantly higher by approximately 2.8–7.6 cm in favor of the exercise group. This magnitude of difference in improvement between both groups was clinically significant due to the large effect size reported (Cohen’s d ranged from 0.52 to 0.78). Our findings are in agreement with those reported in a systematic review in which they found a moderate effect size of physical activity on balance [9]. In addition, our findings are similar to those reported by Granacher et al. (2011); however, we had a larger effect size as compared to Granacher’s study. A possible explanation of the differences in the amount of the effect size reported could be due to the differences in the studies’ population. They recruited sedentary workforce participants in their study [23].

The improvements in balance seen in the exercise group can be attributed to the effect of the exercise program prescribed in this study. The prescribed three sessions weekly dosage of exercise might have enhanced participants’ strength and balance. Our findings are in alignment with those reported by Toeman et al. (2004) who found that strength and balance gains were attained when exercising three times per week in the middle-aged females [24].

Lower limb muscle weakness is considered a fall predictor [19], thus the original OEP focuses on improving lower limb strength that usually reduces with aging. Lee et al. (2014) noted that overall hip muscle strength tends to reduce before women turn the age of 50 years [19]. In addition, mediolateral balance tends to decline in females between 40 to 60 years of age [25]. Improving mediolateral balance might assist in reducing sideways falls in this population. Previous studies have reported a strong link between hip muscles strength and activity and star excursion balance test performance (SEBT) in PM and PL directions [16]. Jaber et al. (2018) reported that reduced hip muscles activity affects the body’s ability to maintain balance, which could have negative consequences on functional performance [16]. Additionally, hip muscles are responsible for corrections of any large balance errors, whereas ankle muscles are accountable for small error corrections [16]. If the motor control of the hip muscles is diminished, the risk of injury might increase [16]. In our study, strengthening hip and ankle muscles was part of the exercise protocol. Strengthening hip musculature is essential to pelvic stability and it is evident when practicing on SEBT and YBT [16]. Moving in the three directions in these tests requires involvement of different lower limb and trunk muscles to hold the pelvis stable [10]. Moreover, when leaning forward and backward the demand on these muscles increases as gravity plays a role on the upper body [10].

Single-leg standing balance has been shown to have a slight to moderate relation with risk of fall in older adults who age 65–94 [7]. As performance in the single leg balance position worsens, the risk of falls usually increases [7]. Balance usually decreases as the person ages, thus addressing the problem early might have positive outcomes. In our study, the one-minute single-leg standing exercise was used as a form of balance training, and we sought to increase the time from 30 seconds to 1 minute in our modified version of OEP as a way to increase the volume and intensity of the exercise to better fit the muscle performance and posture systems of middle-aged adults. This balance exercise was of especially great importance because single-leg activities are critical functional movements [16].

In this study we focused on improving participants’ balance in a hope of preventing them from future falls. It was noted that exercises like squats and single-leg standing enhance central nervous system (CNS) plasticity and improve the function of the balance feedback loop over other balance systems in adults aged 60 years and over [26]. Moreover, such exercises might provide improvement in muscular strength via neuromuscular adaptations [27]. Aging reduces joint position sense, which is part of proprioception in lower limb knee and ankle joints [12]. Therefore, both joints were addressed in the given exercise protocol. Strengthening exercise plays a vital role in enhancing proprioception through the improvement in movement control [12]. Additionally, it was stated that regular physical activity might aid in the preservation of proprioception in older adults [12]. An added factor we believe that might have contributed to the participants’ balance improvements is their compliance with the prescribed exercise program.

Following the eight weeks of training, dynamic balance significantly improved in this study sample. This study illustrates how relatively healthy and active middle-aged adults can benefit from performing comprehensive multimodal strengthening and balance exercises that challenges muscle performance and postural systems.

## Limitations

The limitations of this study were the following: with the participants being mostly females, the results might not represent the middle-aged adult population in full. Furthermore, no blinding to the group allocation or the results was provided to the investigator. Participants’ ankle dorsiflexion range of motion that contributes to anterior reach scores was not tested in this study. Since muscle strength was not measured pre and post intervention, improvements in balance may not necessarily equate to improvement in strength.

## Clinical implications

The outcomes of this study have provided awareness regarding the importance of balance and strength exercises for middle-aged adults. Clinicians need to continue to advise healthy middle-aged adults to exercise regularly using simple programs like the routine prescribed in this study in addition to their usual exercise routines. Moreover, clinicians might need to use YBT more often as it appeared to be sensitive in detecting balance changes post interventions.

## Conclusion

Based on the current work, balance and strengthening exercises appear to be vital to the middle-aged population to help prevent them from future falls. A modified version of Otago Exercise Program is shown to be effective in improving balance in middle-aged population. Moreover, the YBT seems to be a safe dynamic balance assessment tool that can be administered to healthy middle-aged adults. It is a portable and affordable device that can provide quick and accurate information to clinicians about their clients’ balance. Future studies need to explore the sensitivity of the YBT in identifying middle-aged athletes who are at risk of lower limb injury while practicing different sports. Likewise, interventional studies with similar functional training as well as different exercise programs need to be implemented on the same population with examination of the efficacy of the YBT in detecting pre vs post status.

## Supporting information

S1 Checklist CONSORT 2010 checklist of information to include when reporting a randomised trial. (PDF)
